# Metabolic syndrome and benign prostatic hyperplasia: association or coincidence?

**DOI:** 10.1186/s13098-015-0089-1

**Published:** 2015-10-29

**Authors:** Aleksandra Rył, Iwona Rotter, Tomasz Miazgowski, Marcin Słojewski, Barbara Dołęgowska, Anna Lubkowska, Maria Laszczyńska

**Affiliations:** Chair and Department of Histology and Developmental Biology, Pomeranian Medical University, Szczecin, Poland; Department of Medical Rehabilitation, Pomeranian Medical University, Szczecin, Poland; Department of Hypertension and Internal Medicine, Pomeranian Medical University, Szczecin, Poland; Department of Urology and Urological Oncology, Pomeranian Medical University, Szczecin, Poland; Department of Laboratory Diagnostics and Molecular Medicine, Pomeranian Medical University, Szczecin, Poland; Department of Physical Medicine and Functional Diagnostics, Pomeranian Medical University, Szczecin, Poland

**Keywords:** Benign prostatic hyperplasia, Metabolic syndrome, Hormones

## Abstract

**Background:**

It has been suggested that individuals with metabolic syndrome (MetS) may be prone to developing benign prostatic hyperplasia (BPH), but the direction of causality remains uncertain. The objective of this cross-sectional study was to evaluate the association between BPH and MetS in men who were referred to surgery for BPH. We were interested in identifying the anthropometric, metabolic, and hormonal factors that potentially influence the risk of both conditions.

**Methods:**

The study was conducted on 128 males with BPH and 141 without BPH (the control group). Fasting glucose, insulin, lipid profiles, total and free testosterone, estradiol, sex-hormone binding protein (SHBG), dehydroepiandrosterone sulfate (DHEA-S), homeostasis model assessment (HOMA-IR) index, and lipid accumulation product (LAP) were all evaluated.

**Results:**

The prevalence of MetS was higher in patients with BPH than in the controls (58 vs. 41 %; P = 0.007). In comparison to the controls, patients with BPH had higher levels of cholesterol, low density lipoproteins, DHEA-S, insulin, and HOMA-IR, but lower levels of high-density lipoproteins (HDL), estradiol, and SHBG. The significant predictors of BPH were MetS (OR = 1.961), age (OR = 0.11), HDL (OR = 0.91), insulin (OR = 1.224), SHBG (OR = 0.98), and estradiol (OR = 0.978). Waist circumference and LAP inversely correlated with total and free testosterone and SHBG.

**Conclusions:**

Our study confirmed the frequent coexistence of MetS and BPH. This association seems to be a consequence of the MetS-related metabolic derangements, changes in the sex-hormone milieu, and lowered SHBG levels.

## Background

Benign prostatic hyperplasia (BPH) is the most common urological condition among elderly males, affecting approximately half of men over 80 years of age. It usually begins as a simple micronodular hyperplasia with a subsequent macroscopic nodular enlargement that may result in bladder outlet obstruction and the development of lower urinary tract symptoms (LUTS) [1]. Although many inflammatory, hormonal, lifestyle, and environmental factors that may predispose to BPH have been identified, the molecular and stromal mechanisms involved in the pathogenesis of this condition have not yet been fully elucidated. It has been suggested that certain sex-hormone environments—including lower androgen levels and higher estrogen levels—may contribute to the development of BPH [2, 3], as estrogens promote the androgen effects, leading to increase in prostate weight [4]. On the other hand, other studies have shown an opposite, positive association between testosterone level and the severity of LUTS, [5] or no relationship between circulating sex hormones and urological symptoms [6]. Much less is known about the association between BPH and the level of sex-hormone binding globulin (SHBG); to date, studies have yielded inconsistent results. Although some studies have failed to find an association between SHBG levels and the incidence of BPH or the severity of LUTS [7, 8], other reports have demonstrated that SHBG is inversely associated with larger prostate glands [9, 10].

More recently, it has been suggested that individuals with metabolic syndrome (MetS) or its individual components—including central obesity, hyperinsulinemia, insulin resistance, and dyslipidemia—also may be prone to developing BPH and LUTS [3, 11, 12]. On the other hand, some earlier studies found no such relationship [13, 14].

The objectives of the current study were (1) to evaluate the association between BPH and MetS, as defined by the International Diabetes Federation (IDF) diagnostic criteria, and (2) to identify anthropometric, metabolic, and hormonal factors that potentially influence the risk of both conditions.

## Methods

### Study population

The study group consisted of males aged 50–75 years who had been referred to the university-affiliated Department of Urology in Szczecin due to moderate-to-severe LUTS, including filling, irritative, voiding, and obstructive symptoms. Using the standard International Prostate Symptom Score (IPSS) tool, a quality of life questionnaire, and prostate imaging, patients were diagnosed; those receiving a diagnosis of BPH were referred to transurethral resection of the prostate (TURP), according to current recommendations and practical guidelines [15]. Eligibility criteria were, according to the European Association of Urology [16], included in prostate volume above 20–80 ml on transrectal ultrasonography and IPSS result 8 or more.

The control group consisted of age-matched men who had IPSS scores between 0 and 7 and who were neither diagnosed nor considered for treatment for BPH. These subjects were randomly selected from the local general practitioner registers. The exclusion criteria in both groups included a body mass index above 35.0 kg/m^2^, malignancy in the past 5 years, heart failure, severe liver diseases, and chronic kidney disease. Overall, there were 128 males with BPH and 141 without BPH who completed all the study procedures. Informed consent was obtained from each patient in the study. The study protocol was approved by the Ethics Committee of the Pomeranian Medical University in Szczecin and conformed to the ethical guidelines of the 1975 Declaration of Helsinki (6th revision, 2008).

### Measurements

For all study participants, the weight, height, waist circumference (WC), and sitting blood pressure were measured. The body mass index (BMI) was then calculated from the weight and height. Using automated methods and commercially available assays, we measured fasting glucose, insulin, lipid profile (including triglycerides, total cholesterol and high (HDL) and low-density (LDL) lipoproteins), luteinizing hormone (LH), total testosterone (reference ranges in men 8–11, 8 nmol/l) and free testosterone, estradiol, SHBG, and dehydroepiandrosterone sulfate (DHEA-S). Insulin resistance was evaluated by the homeostasis model assessment (HOMA-IR) index, calculated as blood glucose (mmol/l^−1^) × insulin concentration (µIU/ml^−1^)/22.5 [17]. We used the HOMA-IR cut-off of 2.77 to identify subjects with insulin resistance [15, 18]. The lipid accumulation product (LAP) was calculated using the following equation: LAP = (waist circumference in centimeters − 65) × triglycerides (nmol/l). LAP, an estimate of lipid accumulation in adults, has been shown to be a marker of metabolic risk and is a useful tool for stratifying the risk of unfavorable obesity-related outcomes [19, 20].

MetS was defined using the IDF criteria as the presence of central obesity (WC ≥ 94 cm) and any two of the following components: (1) HDL < 1.03 mmol/l or specific treatment for this lipid abnormality; (2) systolic blood pressure ≥ 130, diastolic blood pressure ≥ 85 mm Hg, or treatment of previously diagnosed hypertension; (3) fasting plasma glucose ≥ 5.55 mmol/l or previously diagnosed type-2 diabetes; and (4) triglyceride level ≥ 1.71 mmol/l or specific treatment for this lipid abnormality [21].

### Statistical analysis

The descriptive statistics included frequency distributions (number with condition and percentage) for categorical variables and means, standard deviation (SD), and range for continuous variables. Differences among groups were evaluated by an independent *t*-test or nonparametric Mann–Whitney *U*-test for continuous variables, and by Chi-square test with Yates’ correction for dichotomous variables. Univariate and multiple regression analyses were used to evaluate the independent predictors of MetS and BPH. The relationship between pairs of quantitative variables was represented using Spearman’s rank correlation coefficient. Data were considered to be significant at P < 0.05.

## Results

The baseline anthropometric, metabolic, and hormonal characteristics of the study population are shown in Table 1. Compared to the controls, patients with BPH were of similar age and had similar BMI, WC, and LAP values; had higher levels of total cholesterol and its LDL fraction; but had significantly lower HDL cholesterol. They also had significantly higher fasting insulin concentration and calculated HOMA-IR value. However, when we excluded cases with overt diabetes and those taking hypoglycemic treatments, the percentage of subjects with HOMA-IR > 2.77 was higher in men without BPH than in those with BPH (62 vs. 45 %; P = 0.014). In both men with BPH and those without it, the mean total and free testosterone levels were below the reference ranges, but comparable between the groups.

**Table 1:** Baseline characteristics of study participants

Continuous variables	BPH (n = 128)			Controls (n = 141)			P
	Mean	SD	Range	Mean	SD	Range	
Age (years)	65.76	6.47	52–77	63.66	5.64	50–74	0.081
Weight (kg)	81.37	11.36	61.5–115	81.78	10.20	54–108	0.684
Height (m)	1.73	0.07	1.53–1.98	1.74	0.05	1.58–1.88	0.117
Body mass index (kg/m^2^)	27.05	3.27	18.7–34.7	26.78	2.90	20.0–34.7	0.724
Waist (m)	0.98	0.08	0.79–1.20	0.99	0.086	0.75–1.18	0.492
LAP	56.24	35.90	11.4–296.7	51.98	38.66	7.3–234.9	0.350
Cholesterol (mmol/l)	5.68	1.55	2.16–10.76	5.00	1.57	2.17–9.13	0.001
HDL (mmol/l)	0.92	0.27	0.54–1.94	1.32	0.35	0.50–2.45	0.001
LDL (mmol/l)	3.67	1.49	0.58–8.84	3.35	1.50	0.47–10.49	0.019
Triglycerides (mmol/l)	1.64	0.75	0.66–5.44	1.49	0.84	0.43–4.53	0.132
Glucose (mmol/l)	5.85	0.97	4.27–10.21	6.02	1.20	2.93–10.70	0.489
Insulin (pmol/l)	104.10	43.62	2.64–454.92	81.06	37.32	13.98–185.70	0.041
HOMA-IR	5.00	5.85	0.1–26.28	3.64	1.91	0.10–8.85	0.028
Total testosterone (nmol/l)	14.35	6.45	0.35–29.26	13.83	5.96	0.35–34.08	0.205
Free testosterone (pmol/l)	382.93	250.77	3.09–1272.47	373.92	199.84	36.75–1094.19	0.746
Estradiol (pmol/l)	106.06	75.59	21.92–421.80	146.91	83.96	36.16–441.25	0.001
DHEA-S (μmol/l)	3.34	1.98	0.33–9.34	3.94	2.44	0.03–12.51	0.026
SHBG (nmol/l)	38.17	18.83	2.58–98.0	48.32	25.62	4.93–128.0	0.001
LH (mIU/ml)	10.74	7.16	0.70–67.98	8.04	4.04	1.08–33.09	0.001

Categorical variables	BPH (n = 128)			Controls (n = 141)			P
	n	%		n	%		
Waist > 94 cm	89	69		104	74		0.489
BMI > 25.0 kg/m^2^	103	73		91	70		0.717
Blood glucose > 5.55 mmol/l^a^	77	60		93	66		0.376
HDL < 1.03 mmol/l^a^	46	36		56	40		0.332
Triglycerides > 1.71 mmol/l^a^	74	58		58	41		0.013
Blood pressure > 130/85 mm Hg^a^	76	59		81	57		0.844
Metabolic syndrome	74	58		58	41		0.007

^a^or specific treatment for previously diagnosed condition

The subjects with BPH had higher DHEA-S and LH but lower estradiol and SHBG. In both groups, there were high prevalences of overweight or obesity (BMI above 25.0 kg/m^2^) and of abdominal obesity (waist circumference > 94 cm). The overall prevalence of overweight in all the males participating in the study was 72 %; the value was similar for abdominal obesity. Importantly, despite the similar frequency in both groups of each of the individual components of MetS, the prevalence of MetS was significantly higher in patients with BPH (58 vs. 41 %; p = 0.007).

As expected, when we analyzed the patients with BPH in subgroups with and without MetS (Table 2), those with MetS showed significantly higher BMI, WC, LAP, insulin concentration, and HOMA-IR, as well as higher frequencies of the majority of individual MetS components. Despite similar frequencies in both groups of the subjects with HDL cholesterol levels below 40 mg/dl, patients with MetS had significantly lower mean HDL levels. On the other hand, they had lower SHBG and total (though not free) testosterone. Moreover, in the subgroup with MetS, as many as 88 % of subjects were overweight or obese.

**Table 2:** Anthropometric, metabolic and hormonal characteristics of subjects with and without metabolic syndrome (patients with BPH)

Continuous variables	Metabolic syndrome (n = 74)			Without metabolic syndrome (n = 54)			P
	Mean	SD	Range	Mean	SD	Range	
Age (years)	65.68	6.29	55–77	65.89	6.88	55–77	0.912
Weight (kg)	85.59	10.55	64–115	75.37	9.71	51.5–98	0.001
Height (m)	1.73	0.074	1.54–1.98	1.74	0.071	1.53–1.90	0.996
Body mass index (kg/m^2^)	28.47	2.87	23.5–34.7	25.03	2.72	18.7–34.6	0.001
LAP	70.97	39.48	20.2–296.7	36.25	15.91	11.4–89.9	0.001
Cholesterol (mmol/l)	4.80	1.48	2.17–9.13	5.26	1.66	3.08–11.76	0.121
LDL (mmol/l)	3.14	1.36	0.47–7.43	3.63	1.65	1.01–10.48	0.112
Insulin (pmol/l)	116.82	105.60	11.22–454.80	93.30	101.64	2.64–423.00	0.272
HOMA-IR	5.13	3.63	0.48–26.28	3.99	2.90	0.1–22.47	0.043
Total testosterone (nmol/l)	13.31	6.48	1.04–28.43	15.71	6.24	0.35–29.12	0.020
Free testosterone (pmol/l)	354.92	236.38	0.35–1274.47	420.65	266.26	3.12–1177.27	0.151
Estradiol (pmol/l)	95.08	70.45	22.25–421.80	120.30	80.21	21.92–321.95	0.090
DHEA-S (μmol/l)	3.47	2.12	0.35–9.34	3.12	1.76	0.33–7.19	0.501
SHBG (nmol/l)	35.39	19.75	2.74–98.0	41.89	17.02	2.58–90.3	0.012
LH (mIU/ml)	11.38	8.61	0.70–67.98	9.89	4.52	1.57–23.0	0.211

In both study groups combined, age, insulin, and LH were associated with an increased risk of BPH, and SHBG, estradiol, and HDL with a decreased risk of BPH (Table 3). Importantly, MetS was a robust predictor of BPH (OR = 1.961; 95 % CI 1.207–3.186; P = 0.009). However, when we included MetS in the regression models, together with BMI, age, and LAP as covariates, the association between BPH and MetS was not significant. The presence of BPH was positively associated with LH (β = 0.231; P = 0.001), free testosterone (β = 0.257; P = 0.009), and insulin (β = 0.148; P = 0.025) and negatively with SHBG (β = −0.266; P = 0.001), estradiol (β = −0.227; P = 0.003), and DHEA-S (β = −0.162; P = 0.030).

**Table 3:** Prediction of prevalent BPH by age and metabolic and hormonal factors

	OR	95 % CI	P value
Age (years)	1.110	1.050; 1.174	0.001
Body mass index (kg/m^2^)	1.032	0.420; 2.485	0.944
Waist (m)	0.974	0.884; 1.074	0.598
LAP	0.970	0.921; 1.021	0.243
HDL (mmol/l)	0.910	0.874; 0.948	0.001
LDL (mmol/l)	1.006	0.976; 1.038	0.688
Triglycerides (mmol/l)	1.013	0.991; 1.035	0.247
Insulin (pmol/l))	1.224	1.089; 1.375	0.001
LH (mIU/ml)	1.204	1.091; 1.329	0.001
SHBG (nmol/l)	0.980	0.961; 0.999	0.035
Estradiol (pmol/l)	0.978	0.960; 0.997	0.023
Total testosterone (nmol/l)	1.154	0.938; 1.419	0.176
Free testosterone (pmol/l)	0.999	0.993; 1.004	0.703

*OR* odds ratio

The correlations between anthropometric measurements, LAP, lipid profiles, and sex hormones in patients with BPH and MetS are shown in Table 4. Both waist circumference and the LAP index are inversely correlated with total and free testosterone and SHBG. Total cholesterol and LDL correlated positively with SHBG, while triglycerides correlated negatively with total testosterone and were closely significant with free testosterone. The association between HDL and SHBG bordered on a statistically significant value (P = 0.051).

**Table 4:** Correlations between anthropometric measurements, LAP, lipid profiles and sex hormones in patients with BPH and MetS

	Total testosterone (nmol/l)		Free testosterone (pmol/l)		SHBG (nmol/l)		DHEA-S (μmol/l)		Estradiol (pmol/l)	
	R	P	R	P	R	P	R	P	R	P
BMI (kg/m^2^)	−0.114	0.339	−0.095	0.422	−0.221	0.063	0.035	0.769	0.014	0.907
Waist (m)	−0.283	*0.016*	−0.248	*0.033*	−0.272	*0.021*	−0.023	0.849	−0.086	0.481
LAP	−0.361	*0.002*	−0.286	*0.014*	−0.283	*0.016*	0.132	0.269	−0.149	0.217
Total cholesterol (mmol/l)	−0.072	0.548	0.016	0.895	−0.123	0.302	0.295	*0.012*	−0.130	0.285
LDL (mmol/l)	−0.038	0.752	0.102	0.392	−0.117	0.329	0.280	*0.017*	−0.119	0.327
HDL (mmol/l)	0.194	0.103	0.037	0.755	0.231	0.051	0.024	0.842	0.025	0.839
Triglycerides (mmol/l)	−0.272	*0.021*	−0.210	0.075	−0.201	0.090	0.155	0.193	−0.101	0.404

Significant correlations are shown in italics

*R* Spearman rank correlation coefficient

## Discussion

In this cross-sectional study, we found a high prevalence of MetS and its individual components among patients with BPH who were referred for TURP. In these cases, we found that age and levels of HDL cholesterol, fasting insulin, SHBG, LH, DHEA-S, and estradiol were significantly associated with BPH. Additionally, MetS was a robust single predictor of BPH. The latter finding is consistent with many previous reports suggesting that an association between BPH and MetS may be plausible [11, 12, 22–25], although it is not yet fully understood.

Recently, Vignozzi et al. [26] proposed an interesting three-hit hypothesis on the development of BPH, which may also be helpful in understating the mutual relationship between BPH and MetS. According to this hypothesis, an overt or subclinical inflammation (first hit) could be autosustained or overlapped by metabolic alternations (second hit) and changes in sex-hormone levels (third hit). The combined effects of these may result in overexpression of toll-like receptors, transformation of prostatic cells into antigen-presenting cells, and up-regulation of growth factors (andromedins), leading to prostate enlargement. Among hormonal determinants of BPH, the majority of studies have reported sex steroid imbalances between total or free testosterone, dihydrotestosterone, estrogen, and progesterone levels [2–7], although their circulating levels did not necessarily reflect the causal relationship with prostatic volume and severity of LUTS.

However, meta-analysis [27] of epidemiological evidences between MetS and LUTS secondary to benign prostatic hyperplasia did not demonstrate significant differences of IPSS, IPSS-voiding and IPSS-storage by men with or without MetS. Presence of MetS was not significantly associated with moderate-to-severe LUTS (odds ratio  =  1.13; P  =  0.53) and only altered serum triglycerides and diabetes were associated with this risk.

In addition to these sex-steroid derangements, our results suggest that they may be also extended by changes in circulating DHEA-S and SHBG. Only a few studies in the literature have evaluated SHBG in patients with BPH, and the results have been inconsistent [7–10]. In our series, SHBG concentration was negatively associated with the prevalence of BPH. Moreover, in comparison to the controls, in cases with BPH there were significantly higher levels of DHEA-S and lower levels of SHBG. However, the clinical usefulness of SHBG as a marker of BPH risk seems uncertain, because its serum concentration, like testosterone, is greatly influenced by aging and coexisting obesity [28]. Aside from BPH, it also has been postulated that low SHBG concentrations might be the primary determinant of the incidence of MetS [29]. Indeed, a similar conclusion is suggested by our study. We observed lower SHBG levels in subjects with BPH and coexisting MetS than in those without MetS; however, SHBG negatively correlated with BMI, LAP, and waist circumference, suggesting that its low concentration may be influenced by increased body size, unfavorable fat distribution, and lipid accumulation. This, in turn, may support the third hit in the concept of metabolic alternations in the pathogenesis of BPH [26]. Accordingly, numerous observational data indicate a close link between central obesity and an increased risk of BPH. Giovannucci et al. [30] studied more than 25,000 men and determined that men with WC > 109 cm were 100 % more likely to present LUTS and 38 % more likely to require surgery for BPH, as compared to those with waist circumferences < 109 cm. Likewise, data from the National Health and Nutrition Examination Survey III (NHANES III) showed an association between waist circumference and LUTS, suggesting that progressive prostate enlargement in obese men could induce the occurrence of urinary symptoms [2].

Waist circumference, a major component of MetS, is a surrogate measure of visceral adipose tissue, the accumulation of which is associated with a cluster of metabolic abnormalities, including impaired glucose tolerance, insulin resistance, hypertension, and unfavorable lipid profiles, all of which are risk factors for type-2 diabetes and cardiovascular disease. LAP, calculated from WC and serum triglyceride levels, possesses similar diagnostic and predictive value [19, 20, 31]. Unexpectedly in this study, neither BMI nor waist circumference were determinants of the prevalence of BPH; however, among the studied men with and without BPH, we found a high proportion of men who were obese or centrally obese (72 %), which is likely to have had an impact on our results.

Among other metabolic alternations in males with BPH, we observed higher levels of total and LDL cholesterol, lower HDL cholesterol, and the higher prevalence of increased triglycerides above 1.71 mmol/l than in those without BPH. Moreover, these subjects also had significantly higher insulin levels and HOMA-IR values. From previous studies, it can be determined that MetS—and in particular abnormal lipid profiles including hypertriglyceridemia, low HDL cholesterol, and up-regulation of LDL cholesterol—could induce or maintain an inflammatory state within the prostate [26, 32, 33]. This process could be exacerbated by changes in sex-hormone levels (as observed in our study), such as relative hyperestrogenism, decreases in SHBG levels, and androgen deficiency—that is, medical conditions commonly associated with MetS, and in particular with abdominal obesity. In addition, subjects with excess visceral fat are often prone to developing insulin resistance which, in turn, may be an independent risk factor for BPH [34–36]. Hence, obese, dyslipidemic, and aged males are at risk for MetS, whose components are important determinants of prostate enlargement [7, 11] as well as of other abdominal obesity-related comorbidities, including cardiovascular disease, obstructive sleep apnea, and nonalcoholic fatty liver disease [37].

Our study had some limitations. First, due its observational nature, the associations observed between MetS and BPH may not be causal, on account of some unknown confounding or reverse causality. Second, there was an overexpression in our sample of overweight or obese subjects, which likely overestimated the true prevalence of MetS. Therefore, our results may not apply to the general population of men aged over 50—in particular to normal weight and underweight individuals. Finally, we did not collect data on diet, smoking, or alcohol consumption—i.e., on the modifiable independent factors that can significantly influence the risks of MetS and BPH.

In conclusion, our study confirmed the frequent coexistence of MetS and BPH. This association seems to be a consequence of the MetS-related changes in the sex-hormone milieu and metabolic derangements. Therefore, as obesity, diet (excess consumption of alcohol, red meat, and fat), and a sedentary lifestyle strongly predispose to the development of BPH [4, 38], the broad promotion of a healthy lifestyle and of healthy dietary patterns may be an important tool in both the prevention and treatment of this condition.
